# Chronic Diarrhea and Skin Hyperpigmentation: A New Association

**DOI:** 10.4103/1319-3767.41742

**Published:** 2008-10

**Authors:** Khaled Al Qoaer, Ali Al Mehaidib, Sohail Shabib, Mohammed Banemai

**Affiliations:** 1 Department of Pediatric, Division of Pediatric Gastroenterology, University of Alberta, Edmonton, Canada; 2 Department of Pediatrics, Section of Gastroenterology, King Faisal Specialist Hospital and Research Center, Riyadh, Saudi Arabia

**Keywords:** Diarrhea syndrome, phenotypic diarrhea, infantile diarrhea

## Abstract

**Background/Aims:**

The objective of this study was to describe patients with chronic diarrhea and abnormal skin hyperpigmentation with distinct distribution.

**Methods:**

This is a retrospective review of children who presented with diarrhea and skin hyperpigmentation. The clinical presentation, laboratory investigations as well as endoscopic and histological data were reviewed.

**Results:**

Seven patients with chronic diarrhea had abnormal skin hyperpigmentation with distinct distribution and presented in the first two months of life. Six patients had other features such as abnormal hair and facial dysmorphism. Mental retardation was reported in one patient. Consanguinity was positive in six patients, and there was family history of consanguinity in four patients, with two patients being siblings. No significant immunodeficiency was reported. Intestinal biopsies were obtained in six patients and showed active chronic inflammation in three patients, partial villous atrophy in two patients, and eosinophilic infiltrate with mild villous atrophy in one patient. Colonic biopsies showed mild focal colitis in three patients and mild colitis with eosinophilic infiltrate in one patient. Skin biopsies showed a greater number of melanophagies with fibrosis of papillary derma in two patients but skin biopsy was normal in one patient. The hair of two patients was analyzed by electron microscopy, which showed an abnormal pattern with decreased pigmentation and diameter; however, its chemical analysis was normal. Two other patients had trichorrhexis nodosa, but no abnormalities were seen in one patient. Chromosomal number was normal in three patients. One patient died because of sepsis, and only one patient was dependent on total parenteral nutrition.

**Conclusions:**

We believe that this association might represent a new syndrome with an autosomal recessive inheritance that warrants further studies.

The underlying causes of chronic diarrhea beginning early in life are well defined. Infectious and postinfectious enteropathies and food-sensitive/allergic enteropathy account for the majority of cases. However, the etiology remains unknown in about 15–20% of the cases.[1]

Although chronic diarrhea has been reported in association with phenotypic abnormalities, we observed an interesting association between chronic diarrhea and hyperpigmented skin lesions in addition to some other clinical features, which, to the best of our knowledge, have not been described before.

## PATIENTS AND METHODS

Among children with chronic diarrhea who presented to King Faisal Specialist Hospital and Research Center in Riyadh, Saudi Arabia, between 1990 and 2005, seven patients were found to have consistent, abnormal skin lesions. We retrospectively reviewed the records of these children and looked at the clinical, biochemical, and histopathological findings.

We reviewed all investigations including blood, urine, and stool looking for the underlying cause(s) of diarrhea. The immunological workup included analyses of lymphocyte populations with T- and B-cell-specific antibodies, lymphocyte markers, blastogenesis, and immunoglobulin levels. Metabolic screening included the analyses of serum amino acid and urinary organic acid levels. Chromosomal analyses were reviewed. The histopathology of gastrointestinal, liver, skin, and hair biopsies was also reviewed.

A computer-based technique was used for the review of current literature. Original articles were identified from the MEDLINE database via PubMed and Ovid for all reports of infants or children with chronic diarrhea and hyperpigmentation. Another combination of the terms: café au lait spots, skin lesions, hair abnormalities, and diarrhea was used in the search.

## RESULTS

Clinical features are summarized in Tables 1 and 2. Seven patients were included in this retrospective study (three male and four females). One patient was from Tunisia, and the others were Saudi. All but one (86%) were born at term; three (43%) were small for their gestational age. Six patients (86%) were products of marriages between cousins. Family history of a similar presentation was reported in four patients (57%), and two patients among them (nos. 1 and 2) were siblings. Two other patients had a sibling as well as a cousin with a history of similar disease. All patients presented in the first two months of life ([five (71%) in the first two weeks] with severe watery and nonbloody diarrhea. At the onset of diarrhea, three (43%) patients were exclusively being breastfed, while the others were being fed with a combination of cow's milk formula and breast milk. All patients were admitted at presentation at which point all were failing to thrive.

**Table 1:** Clinical features

Patient no.	Age (years)	Sex	Birth Wt. (kg)	Gestation	Age at onset (days)	TPN duration (months)	Family history	Consanguinity
1	15	F	3.5	T	10	0	Yes	Yes
2	7	M	3.9	T	14	0.5	Yes	Yes
3	3	F	1.6	36 W	14	0	No	Yes
4	4	M	2.6	T	40	0	No	No
5	3.5	M	2.7	T	4	8	Yes	Yes
6	1.5	F	1.8	T	14	0	Yes	Yes
7	3	F	2.2	T	14	38	No	Yes

**Table 2:** Frequency of different clinical features in affected children

Clinical features	n (%)
Diarrhea	7 (100)
Hyperpigmentation	7 (100)
Failure to thrive	7 (100)
Abnormal hair	6 (86)
Facial dysmorphism	6 (86)
Abnormal teeth	2 (28)
Mental retardation	1 (14)
Hyperkeratosis	2 (28)
Goiter	1 (14)

**Figure 1:**
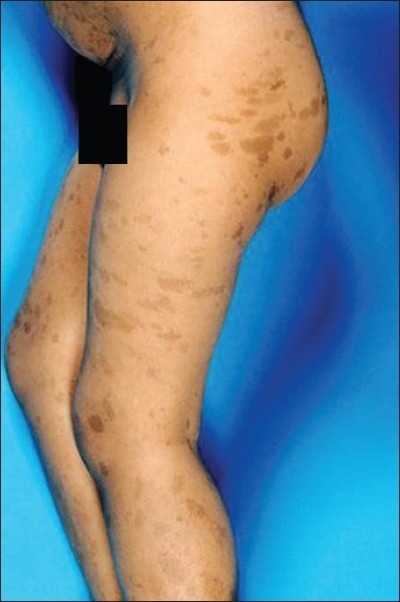
Hyperpigmented skin lesions involving the lower half of the body

The most interesting and striking clinical features that were consistent in all the patients were the skin lesions. These lesions were in the form of scattered tan-brown, hyperpigmented spots or maculae. They were different in sizes (0.5–5 cm in diameter) and number (> 10). These lesions were not raised but had distinct borders and all were located in the lower half of the body (from the waist and below) [Figure 1].

**Figure 2:**
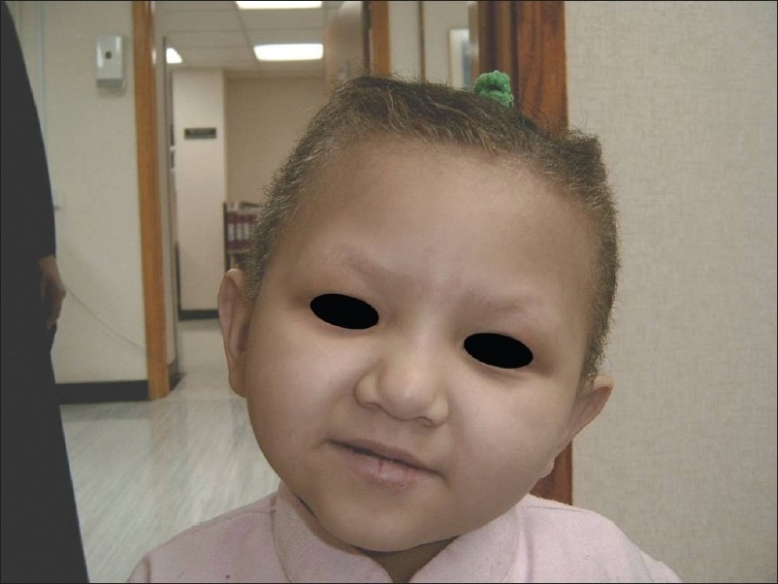
A three year-old girl with an abnormal phenotype in the form of a prominent forehead and cheeks, flat broad nose, and hypertolerism

**Figure 3:**
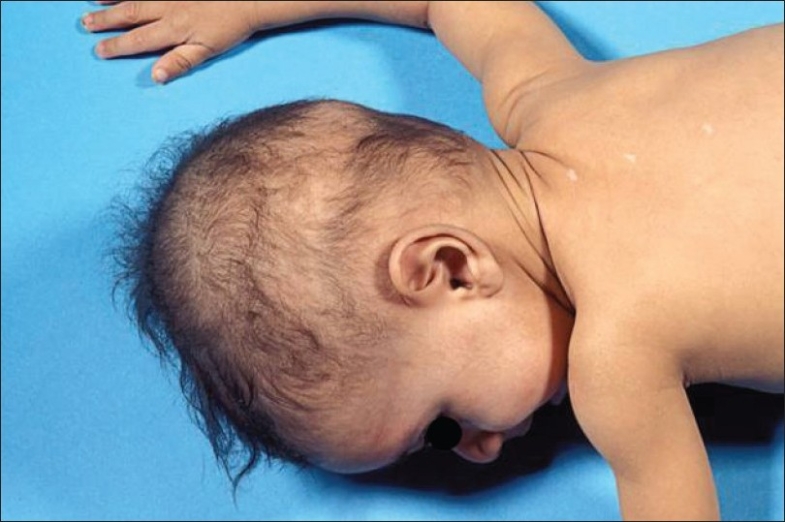
Abnormal sparse, woolly and easily removable scalp hair

Facial dysmorphism and abnormal scalp hair were seen in six patients (86%). Facial abnormalities such as a prominent forehead and cheeks, flat broad nose, and hypertolerism [Figure 2] were observed. They had uncombable, woolly, and easily removable hair [Figure 3]. Two patients (29%) also had peg teeth and hyperkeratosis of both the soles and the palms. Mild mental retardation was reported in one of them (no. 1). She had otherwise normal neurological evaluation including brain MRI; she developed asymptomatic goiter. Thyroid-stimulating hormones and serum thyroid hormones were normal.

In all patients, the diarrhea did not improve with fasting, and investigations including blood, urine, and stool tests did not reveal any specific etiology.

Gastrointestinal biopsies were obtained from six patients. Small bowel biopsies showed normal villi with mild increases in the number of inflammatory cells—plasma cells, lymphocytes, eosinophils, and neutrophils in three patients, and partial villous atrophy with the same picture of inflammation in two patients. Colonic biopsies showed nonspecific, mild colitis.

On the other hand, one patient had partial villous atrophy and eosinophilic infiltration in duodenal, gastric as well as colonic biopsies.

The ultrastructural study did not reveal any microvillus atrophy or tufting enteropathy.

Skin biopsies were obtained from four patients. The results of microscopic examination were unremarkable in two patients and showed increased amount of melanophagies with fibrosis of papillary dermis in the other two patients. The hair of five patients was analyzed by using electron microscopy. It showed an abnormal pattern with decreased shaft diameter in two patients. There was a picture of trichorrhexis nodosa in two other patients, whereas hair examinations were unremarkable in one patient. Karyotyping done for three patients was normal. One patient had persistently elevated liver enzymes, and the liver biopsy showed mild fibrosis with no evidence of cirrhosis. Liver enzymes normalized spontaneously. Another patient had a liver biopsy because of an abnormal lesion seen on ultrasound examination. It only showed evidence of steatosis and no other abnormalities.

All histopathological findings are summarized in Table 3. Apart from central line-related infections, no patient had any recurrent or serious infections. Immunological investigations did not reveal any specific abnormalities. Two patients (29%) had increased total IgA levels, while one patient (14%) had a nonsignificantly increased CD8-T cell count with a low CD4/CD8 ratio. Metabolic screening was unremarkable in all patients. One patient had a transient low serum zinc level, whereas the other six patients had normal levels.

**Table 3:** Histopathological finding

Patient no.	Small bowel biopsies	Colon biopsy	Skin biopsy	Liver biopsy	Hair
1	Normal villi with active chronic inflammation	Not done	Increased number of melanophagies with fibrosis of papillary derms	Slight fibrosis with minimal necrosis	Abnormal pattern with decrease in the shaft diameter
2	Normal villi with active chronic inflammation	Not done	Increased number of melanophagies with fibrosis of papillary dermis	Not done	Abnormal pattern with decrease in the shaft diameter
3	Partial villous atrophy with eosinophilic infiltration	Mild colitis with eosinophilic infiltrate	Normal morphology	Not done	Not done
4	Mild chronic duodenitis with no villous atrophy	Mild colitis	Not done	Not done	Not done
5	Not done	Not done	Not done	Not done	Unremarkable
6	Partial villous atrophy with mild chronic inflammation	Not done	Not done	Not done	Trichorrhexis nodosa
7	Partial villous atrophy	Mild focal colitis	Normal morphology	Steatosis	Trichorrhexis nodosa

Total parenteral nutrition (TPN) was required in four patients (57%) for different lengths of time; only one patient (14%) was dependent on TPN. The remaining patients managed well with elemental formula and/or a gluten-free and cow milk-free diet. The patient with eosinophilic gastroenteritis was treated with systemic glucocorticosteroids and showed a partial response. However, she finally died with sepsis; this was the only death in this series.

## DISCUSSION

Intractable diarrhea with phenotypic abnormalities (prominent forehead and cheeks, a flat, broad nose, hypertelorism, and unusual, woolly, unmanageable hair that came out in tufts) was described by Stankler ***et al.*** in 1982.[2] The syndrome was further defined by Girault ***et al.*** in 1994;[3] a few other cases were reported later.[4–6]

Some other associated abnormalities were described such as low birth weight,[4–6] variable immunodeficiency-like defective antibody responses, defective antigen-specific skin tests and monoclonal hyperimmunoglobulinemia A,[3,5] liver cirrhosis,[6] and neonatal hemochromatosis.[4] Neither café au lait (CAL) spots nor any hyperpigmented lesions were reported in any of these studies and reports.

Our patients showed some similar features such as chronic diarrhea, facial dysmorphism, and hair abnormalities. Consistent with earlier findings, two patients had nonspecific high IgA levels,[3,4] although no clear immunological abnormalities were noted in our patients. The outcome in our study looks favorable, as most of the patients survived and fared satisfactorily without parenteral nutrition. This is an important point as the outcome was poor in all reports mentioned earlier. Family history and the high frequency of consanguinity are the other important and striking features in our patients, which raised the possibility of the autosomal recessive inherited character of this disorder.

The unique and the striking feature in this study is the presence of multiple hyperpigmented skin lesions (café au lait spots) with a special distribution confined to the lower half of the body in all patients.

Although CAL spots are common in the general population, multiple spots may indicate an underlying pathological disorder. The frequency of having at least one CAL spot has been reported in 0.1–3% of newborns while it has been reported to be as high as 27% in older children (< 10 years old). More than three CAL spots have been noted in only 0.2–0.3% of normal schoolchildren. Neurofibromatosis type 1 is the most common disorder associated with CAL spots.[7] Diagnosis of NF1 requires the fulfillment of two or more of National Institutes of Health (NIH) criteria [Table 4]. These criteria have limited utility in young children. Except for CAL spots, most features are not present at early stages of life.[8] However, at one year of age, 75% of the probands familial cases and 50% of sporadic cases of NF1 tend to fulfill at least two of these criteria. At eight years of age, the probands with NF1 have met two or more of the diagnostic criteria, but only 4% of sporadic cases did not.[9] The gastrointestinal tract is rarely involved in this genetic disorder. Altuntos ***et al.*** reported a four-year-old boy with NF1 who died because of chronic diarrhea and severe protein-losing enteropathy.[10] Plexiform neurofibromatosis of the liver and mesentery was found in an 11-year-old boy who had had a history of diarrhea, abdominal pain, and a failure to gain weight for two years.[11]

**Table 4:** NIH consensus criteria for neurofibromatosis

Diagnosis requires two criteria
≥ 6 café au lait spots, ≥ 5 mm diameter in children, ≥ 15 mm in adults
≥ 2 neurofibromas of any type or 1 plexiform neurofibroma
Axillary/inguinal freckling
Optic glioma
≥ 2 Lisch nodules
Distinctive osseous changes (e.g., sphenoid wing dysplasia or pseudoarthrosis)
First-degree relative with neurofibromatosis 1

National Institutes of Health (NIH) consensus statement 1987 Jul 13–15; 6 (12):1–19

Another four-year-old boy with NF1, who had presented with a chronic episodic constipation and diarrhea, was found to have intestinal neuronal dysplasia type B.[12]

In our study, NF1 was unlikely because of a few reasons: First, an abnormal phenotype and hair abnormalities are not features of NF1. Second, the diagnostic criteria were not present in any patient, even in those who had reached puberty. Third, the striking distribution of CAL spots that spared in the upper half of the body in all patients made the diagnosis of NF1 less likely. A review of current literature failed to show any association between our findings and any CAL spot disorder listed in Table 5.

**Table 5:** Conditions with multiple café au lait spots

Tuberous sclerosis	McCune-Albright syndrome
Ataxia telengectasia syndrome	Bloom syndrome
Fanconi anemia	Silver-Russell syndrome
LEOPARD syndrome	Watson syndrome
Noonan syndrome	Familial café au lait spots

Finally, we believe that our patients show a distinct combination of signs which may represent a new disorder or syndrome that requires further investigation.
